# Raloxifene Prevents Skeletal Fragility in Adult Female Zucker Diabetic Sprague-Dawley Rats

**DOI:** 10.1371/journal.pone.0108262

**Published:** 2014-09-22

**Authors:** Kathleen M. Hill Gallant, Maxime A. Gallant, Drew M. Brown, Amy Y. Sato, Justin N. Williams, David B. Burr

**Affiliations:** 1 Department of Anatomy and Cell Biology, Indiana University School of Medicine, Indianapolis, Indiana, United States of America; 2 Department of Nutrition Science, Purdue University, West Lafayette, Indiana, United States of America; Georgia Regents University, United States of America

## Abstract

Fracture risk in type 2 diabetes is increased despite normal or high bone mineral density, implicating poor bone quality as a risk factor. Raloxifene improves bone material and mechanical properties independent of bone mineral density. This study aimed to determine if raloxifene prevents the negative effects of diabetes on skeletal fragility in diabetes-prone rats. Adult Zucker Diabetic Sprague-Dawley (ZDSD) female rats (20-week-old, n = 24) were fed a diabetogenic high-fat diet and were randomized to receive daily subcutaneous injections of raloxifene or vehicle for 12 weeks. Blood glucose was measured weekly and glycated hemoglobin was measured at baseline and 12 weeks. At sacrifice, femora and lumbar vertebrae were harvested for imaging and mechanical testing. Raloxifene-treated rats had a lower incidence of type 2 diabetes compared with vehicle-treated rats. In addition, raloxifene-treated rats had blood glucose levels significantly lower than both diabetic vehicle-treated rats as well as vehicle-treated rats that did not become diabetic. Femoral toughness was greater in raloxifene-treated rats compared with both diabetic and non-diabetic vehicle-treated ZDSD rats, due to greater energy absorption in the post-yield region of the stress-strain curve. Similar differences between groups were observed for the structural (extrinsic) mechanical properties of energy-to-failure, post-yield energy-to-failure, and post-yield displacement. These results show that raloxifene is beneficial in preventing the onset of diabetes and improving bone material properties in the diabetes-prone ZDSD rat. This presents unique therapeutic potential for raloxifene in preserving bone quality in diabetes as well as in diabetes prevention, if these results can be supported by future experimental and clinical studies.

## Introduction

People with type 2 diabetes mellitus have a greater risk for bone fragility fractures compared with healthy adults, despite normal or higher bone mineral density [1]–[5]. This suggests that bone quality, not quantity, is responsible for the increase in fracture risk in diabetes. Raloxifene is a selective estrogen receptor modulator (SERM) used clinically in women to treat post-menopausal osteoporosis. Our group has previously shown that dogs treated with raloxifene have greater femoral and vertebral toughness, despite no significant effect on bone mineral density [6], [7]. Similarly, in post-menopausal women, raloxifene decreases risk of fracture with little effect on bone mineral density [8]–[10]. This indicates that raloxifene improves bone resistance to fracture by affecting bone quality, and may therefore be an agent with potential to improve bone properties in diabetes where fracture risk is higher apparently due to reduced bone quality rather than reduced bone mass.

The Zucker Diabetic Sprague-Dawley (ZDSD) rat is a recently developed rodent model of type 2 diabetes crossbred from the diet-induced-obesity CD (Sprague-Dawley-derived) and lean Zucker Diabetic Fatty rats (ZDF*^fa/+^*) [11]. Unlike the diabetic obese ZDF^fa/fa^ rats, ZDSD rats do not have a leptin receptor mutation, and both sexes develop a type 2 diabetes phenotype of polygenic origin more gradually with age or by induction with a high-fat diet, thus reflecting more closely the pathogenesis of human type 2 diabetes [11], [12]. This study aimed to test the effects of raloxifene on bone quality and strength in adult female ZDSD rats. Although we have shown positive effects of raloxifene on bone material properties in normoglycemic animals, no studies have been performed in a model subject to diabetes to determine whether raloxifene in a hyperglycemic environment will prevent increased skeletal fragility.

## Materials and Methods

### Animals and Experimental Design

Twenty-week-old female (n = 24) Zucker Diabetic Sprague Dawley (ZDSD) rats (PreClinOmics, Indianapolis, IN) were randomized (n = 12/group) to receive daily subcutaneous injections of raloxifene (0.5 mg/kg, Eli Lilly Co., Indianapolis, IN) or vehicle (10% cyclodextrin, Sigma-Aldrich) for 12 weeks, and all rats were fed a diabetogenic high-fat diet (48% fat; 5SCA, TestDiet, Richmond, IN) for the duration of the study. The high-fat diet is used to synchronize diabetes induction. Additionally, in contrast to male ZDSD rats that will develop diabetes with age even while on a normal rat diet[11], female ZDSD rats are more resistant to developing diabetes and require the high-fat diet for diabetes induction and to maintain the diabetic state. Blood glucose was measured weekly by glucometer (AlphaTRAK, Abbott Laboratories, Abbott Park, IL) and diabetes was defined as blood glucose ≥ 250 mg/dL for 2 consecutive weeks. Whole blood and serum samples were collected at baseline and sacrifice. Glycated hemoglobin (HbA1c,%) was measured in whole blood by immunological assay (Daytona Chemistry Anlayzer, Randox Laboratories, Kearneysville, WV). Serum insulin was measured by ELISA (Mercodia Inc., Winston Salem, NC) and serum triglycerides were measured by colorimetric assays (Daytona Chemistry Analyzer, Randox Laboratories, Kearneysville, WV). Serum c-telopeptide of type I collagen was measured by ELISA (Biotang Inc., Lexington, MA). Prior to sacrifice, rats were double-labeled by intraperitoneal injections of calcein (5 mg/kg; Sigma-Alrich, St. Louis, MO) with a 7-day interlabel period and a 3-day period for incorporation and washout (i.e. 1-7-1-3). Bones (femora, lumbar vertebrae) were collected at the time of sacrifice when rats were 32-weeks-old. Femora and L4 vertebrae were wrapped in saline-soaked gauze and frozen at −20°C for storage prior to bone imaging and mechanical testing; L5 vertebrae were cleaned of soft tissue and fixed in 10% phosphate-buffered formalin for 48 h, then transferred to 70% ethanol, dehydrated in a graded series of ethanol from 70–100%, then embedded (undecalcified and unstained) in methyl-methacrylate with 3% dibutyl phthalate (Sigma-Aldrich, St. Louis, MO) for dynamic histomorphometry. This protocol was approved by the Indiana University Animal Care and Use Committee, and all institutional and national guidelines for the care and use of laboratory animals were followed.

### Bone Imaging

Dual-energy x-ray absorptiometry (DXA, GE Lunar PixiMus, Madison, WI) was performed on excised right femora and L4 vertebrae for measures of areal bone mineral density (aBMD g/cm^2^), bone mineral content (BMC, g) and area (cm^2^). Peripheral quantitative computed tomography (pQCT, XCT Research SA+, Stratec Medizintechnik GmbH, Pforzheim, Germany) was performed on the right femur midshaft for cortical bone morphometric properties (volumetric BMD (vBMD), BMC, cortical area and thickness, periostal and endosteal circumferences and x-axis cross-sectional moment of inertia). Micro-computed tomography (µCT, Brucker Skyscan 1172, Kontich, Belgium) was performed on L4 vertebral bodies and the right distal femur for cancellous bone morphometric properties. Scans were done at 8 µm resolution, 65 kV and 120 µA using a 0.7° rotation step. Reconstructed µCT images (NRecon software) were analyzed using CT Analyzer software (Skyscan, Kontich, Belgium). The same parameters/thresholds were used for each site for reconstruction and analysis. Outcome measurements included whole vertebral body bone volume (BV, mm^3^), trabecular bone volume fraction (BV/TV,% [where TV is tissue volume]), trabecular number (Tb.N, mm^−1^), trabecular thickness (Tb.Th, mm), trabecular separation (Tb.Sp, mm), connectivity density (Conn.D, mm^−3^), and structural model index (SMI).

### Mechanical Testing

Mechanical properties of the femur mid-diaphysis were determined by three-point bending using standard methods [13]. Briefly, bones were thawed to room temperature, and placed posterior side down on the bottom support (18 mm wide) of a servohydraulic test system (100P225 Modular Test Machine, TestResources, Shakopee, MN), so that the descending probe contacted the central anterior surface. Bones were loaded to failure using a displacement rate of 2 mm/min. Force vs. displacement data was collected at 10 Hz. Material properties were calculated based on standard equations using structural mechanical properties and geometric measures from pQCT [13]. Reduced platen compression (RPC) was used to determine mechanical properties of cancellous bone on a 2 mm thick slab of distal femur (100P225 Modular Test Machine, TestResources, Shakopee, MN). For RPC, platen size was set at 70% of the maximum circle diameter to include only cancellous bone [6], which was determined by uCT scanning of the samples prior to mechanical testing. Tests were performed at 0.5 mm/min and data collected at 2 Hz until sample failure.

Mechanical properties of L4 vertebrae were determined by axial compression after removal of vertebral processes using a dremel tool with a minisaw attachment, and removal of the cranial and caudal endplates parallel to each other using a low-speed bone saw (Isomet, Buehler, Lake Bluff, IL). L4 vertebral bodies (+/- 3.5 mm height) were loaded at a rate of 0.5 mm/min until failure (100P225 Modular Test Machine) and data were collected at 10 Hz.

Structural mechanical properties of femoral cortical bone, L4 vertebrae and cancellous bone from the RPC testing were determined from the load-deformation curves using standard definitions. Material properties were calculated based on standard equations using structural mechanical properties and geometric measures from caliper measurements and pQCT (cortical bone) or µCT [13].

### Bone Turnover

Bone turnover was measured by serum C-terminal telopeptides of type I collagen (Ctx) by EIA (RatLaps^™^, IDS, Inc.), and by dynamic histomorphometry of L5 vertebrae. Thin sections (approximately 6 µm) of the L5 vertebra were cut longitudinally with a microtome (Reichert-Jung SuperCut). Approximately 5 mm^2^ of cancellous bone tissue 0.5 mm from the endocortical surface was analyzed from one section. Measurements were made at 200x magnification using a fluorescent microscope (Nikon Optiophot 2, Nikon, Inc., Garden City, NY) and images were analyzed using the Bioquant system (R&M Biometrics, Nashville, TN). All measurements and calculations were performed following the guidelines of the American Society for Bone and Mineral Research Histomorphometry Nomenclature Committee [14]. Parameters measured included single-label perimeter (sL.Pm), double-label perimeter (dL.Pm), and interlabel width (Ir.L.Wi). From these primary measurements, the following outcome parameters were calculated: mineral apposition rate (MAR  =  Ir.L.Wi/7 days [µm/day]); mineralizing surface (MS/BS  =  (0.5*sL.Pm + dl.Pm)/B.Pm*100 [%]); and bone formation rate (BFR/BS  =  MAR*MS/BS*365 [µm^3^/µm^2^/year]).

### Statistical analyses

The planned two-way analysis of variance (diabetes and raloxifene as factors) was not possible because none of the raloxifene-treated rats became diabetic. Thus, one-way analysis of variance with Tukey's posthoc analysis was used to detect differences in means among the three groups: raloxifene-treated (RAL), vehicle-treated non-diabetic (VEH-ND), and vehicle-treated diabetic rats (VEH-D). Body weight was tested as a covariate for all measures and used for DXA variables and L4 BV/TV. Diabetes induction was analyzed by log-rank test of Kaplan-Meier survival curves and Fisher's exact tests. Statistical analyses were performed using SAS 9.2 software (Cary, NC) and significance was set at α 0.05. Values are presented as least squares means ± SEM unless otherwise noted.

## Results

**Figure 1:**
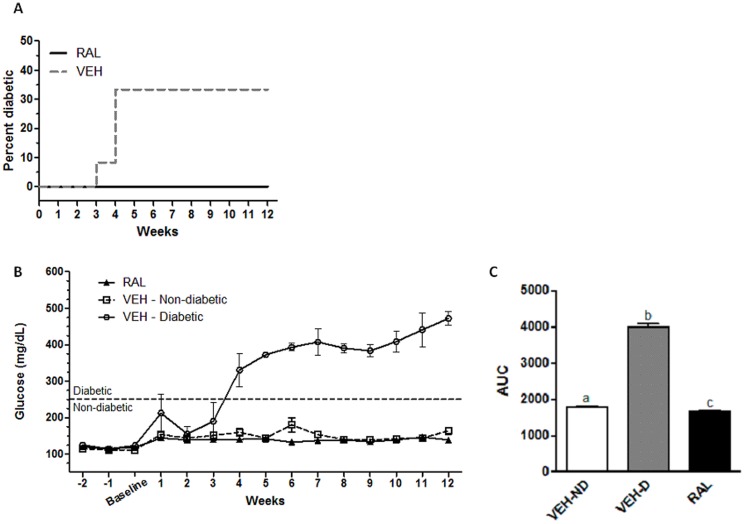
Diabetes Incidence and Glucose Levels in Raloxifene and Vehicle-Treated Rats. Panel A) Female ZDSD rats treated with raloxifene (RAL) had lower incidence of diabetes compared with vehicle treated rats (VEH) by survival analysis (p = 0.03) (shown), but by Fisher's exact test, the frequency of diabetes in VEH and RAL treated rats did not reach statistical significance (p = 0.09). Panels B,C) Over the course of the study, blood glucose was lowest in raloxifene treated rats (RAL), and highest in vehicle-injected rats that became diabetic (VEH-D), as assessed by area-under-the-curve (AUC). Different letters indicate differences in means with p<0.05.

Rats randomized to receive raloxifene injections and vehicle injections had similar baseline body weight (mean ± SD: 342±14 and 348±23 g, respectively), blood glucose (mean ± SD: 114±7 and 112±12 mg/dL, respectively), HbA1c (mean ± SD: 4.5±0.3 [n = 11] and 4.5±0.1% [n = 9], respectively), serum insulin (mean ± SD: 0.50±0.26 [n = 6] and 0.47±0.36 [n = 10], respectively) and serum triglycerides (mean ± SD: 3.37±1.39 [n = 7] and 2.73±1.01 [n = 9]). After 12 weeks, none of the 12 rats treated with raloxifene became diabetic whereas 4 out of 12 rats that received vehicle injections became diabetic. By Fisher's exact test, the difference in diabetes frequency did not reach statistical significance (p = 0.09). However, by survival analysis of Kaplan-Meier curves, raloxifene significantly reduced diabetes induction (p = 0.03) (**Fig. 1a**).

At the time of sacrifice, vehicle-treated non-diabetic (VEH-ND) rats weighed more than both vehicle-treated diabetic (VEH-D) (p<0.0001) and raloxifene-treated (RAL) (p<0.0001) rats (**Table 1**).VEH-D rats had higher blood glucose over the course of the study, as determined by area-under-the-curve (AUC) compared with RAL or VEH-ND rats (p<0.0001) (**Fig. 1b,c**). Additionally, RAL-treated rats had lower cumulative blood glucose over the course of the study (AUC) than VEH-ND rats (p = 0.048) (**Fig. 1c**), but endpoint values were not significantly different between RAL-treated and VEH-ND rats (**Table 1**). At sacrifice, HbA1c was higher in VEH-D (p<0.0001) compared with RAL and VEH-ND rats. Serum insulin tended to be lower in the VEH-D rats compared with the VEH-ND and RAL-treated rats, but this was not significant. Serum triglycerides were higher in VEH-D rats compared with the RAL-treated rats (p = 0.02) (**Table 1**). Bone resorption measured bv serum Ctx was similar among VEH-ND, VEH-D, and RAL-treated rats. However, dynamic histomorphometry showed non-significant trends for lower MS/BS (−28%) and BFR/BS (−26%) but higher MAR (+ 10%) in RAL-treated rats versus VEH-ND rats. Additionally, diabetic rats (VEH-D) had significantly lower MAR and non-significant trends for lower MS/BS and BFR/BS compared to the non-diabetic animals (VEH-ND or RAL-treated) (**Table 1**).

**Table 1:** Body weight, metabolic parameters and bone turnover at end of study^a^.

	VEH-ND (n = 8)		VEH-D (n = 4)^b^		RAL (n = 12)	
Body weight, g	532.5 (10.9)	a	411.0 (15.4)	b	417.8 (8.9)	b
Serum glucose, mg/dL	163.8 (8.0)	a	472.3 (11.3)	b	138.9 (6.5)	a
Blood HbA1c,%	4.8 (0.2)	a	10.3 (0.2)	b	4.7 (0.1)	a
Serum triglycerides, mg/dL	5.7 (0.7)	ab	7.7 (1.0)	a	4.4 (0.6)	b
Serum insulin, µg/L	3.6 (0.6)	a	1.8 (0.9)	a	3.2 (0.5)	a
Serum Ctx, ng/mL	19.6 (5.0)	a	34.9 (7.0)	a	23.9 (4.1)	a
**L5 Histomorphometry**						
MAR, µm/day	1.03 (0.11)	a	0.43 (0.18)	b	1.13 (0.10)	a
MS/BS,%	4.60 (0.85)	a	1.07 (1.39)	a	3.33 (0.76)	a
BFR/BS, µm^3^/µm^2^/year	18.27 (3.78)	a	2.13 (6.18)	a	13.52 (3.38)	a

a Different letters in each row indicate differences among groups by Tukey's post-hoc comparisons, p<0.05.

b n = 3 for VEH-D for the L5 histomophometry measures due to unavailable sample from 1 rat.

Areal bone mineral density and bone mineral content of the whole femur were lower in VEH-D compared with the other two groups (**Table 2**). There were no significant differences among groups for pQCT measures of the femoral midshaft. In the distal femur, bone volume normalized to tissue volume, trabecular thickness, and trabecular number were lower, and trabecular separation was higher in VEH-D rats compared with the other two groups, and structure model index was higher (more rod-like) in VEH-D compared with RAL rats (**Table 2**). There were no significant differences among groups for DXA or µCT measures of L4 vertebrae (**Table 2**).

**Table 2:** Bone mass and microarcitecture of the femur and L4 vertebra from female ZDSD rats^a^.

	VEH-ND (n = 8)		VEH-D (n = 4)		RAL (n = 12)	
**Total Femur DXA**						
aBMD, g/cm^2^	0.249 (0.005)	a	0.224 (0.004)	b	0.246 (0.003)	a
BMC, g	0.626 (0.010)	a	0.574 (0.009)	b	0.619 (0.006)	a
Area, cm^2^	2.51 (0.03)	a	2.56 (0.03)	a	2.51 (0.02)	a
**Midshaft femur pQCT**						
Ct. vBMD, mg/cm^3^	1473 (2)	a	1467 (3)	a	1475 (2)	a
Ct. BMC, mg/mm	10.2 (0.1)	a	10.1 (0.1)	a	10.2 (0.1)	a
Ct.Ar, mm^3^	6.90 (0.06)	a	6.89 (0.08)	a	6.90 (0.05)	a
Ct.Th, mm	0.86 (0.01)	a	0.85 (0.01)	a	0.85 (0.01)	a
Periosteal Circumference, mm	10.7 (0.08)	a	10.9 (0.11)	a	10.7 (0.07)	a
Endosteal Circumference, mm	6.02 (0.08)	a	5.99 (0.11)	a	6.01 (0.06)	a
**Distal Femur µCT**						
BV/TV,%	42.2 (2.2)	a	27.9 (3.2)	b	42.3 (1.8)	a
Tb.Th, mm	0.112 (0.003)	a	0.092 (0.005)	b	0.110 (0.003)	a
Tb.Sp, mm	0.170 (0.008)	a	0.206 (0.011)	b	0.171 (0.006)	a
Tb.N, #	3.75 (0.13)	a	3.02 (0.19)	b	3.83 (0.11)	a
Conn.Dn, #/mm^3^	126.9 (6.4)	a	116.7 (9.1)	a	135.7 (5.3)	a
SMI, units	0.50 (0.20)	ab	1.32 (0.28)	b	0.39 (0.16)	a
**L4 DXA**						
aBMD, g/cm^2^	0.127 (0.004)	a	0.125 (0.003)	a	0.129 (0.002)	a
BMC, g	0.025 (0.002)	a	0.024 (0.002)	a	0.025 (0.001)	a
**L4 µCT**						
BV/TV,%	38.6 (2.5)	a	40.7 (2.2)	a	42.6 (1.5)	a
Tb.Th, mm	0.102 (0.001)	a	0.097 (0.002)	a	0.102 (0.001)	a
Tb.Sp, mm	0.197 (0.008)	a	0.197 (0.011)	a	0.194 (0.006)	a
Tb.N, #	4.04 (0.14)	a	4.06 (0.19)	a	4.08 (0.11)	a
Conn.Dn, #/mm^3^	97.2 (7.1)	a	96.8 (10.0)	a	100.4 (5.8)	a
SMI, units	0.22 (0.10)	a	0.22 (0.14)	a	0.18 (0.08)	a

a Different letters in each row indicate differences among groups by Tukey's post-hoc comparisons, p<0.05.

RAL-treated rats had greater energy to failure and post-yield energy to failure in femoral cortical bone compared with VEH-ND and VEH-D rats (**Table 3**). Correspondingly, the material-level properties of femoral toughness and post-yield toughness were also higher in RAL-treated rats (**Table 3**). There were no differences among groups in structure-level or material-level mechanical properties from vertebral axial compression (**Table 3**). RPC of the distal femur cancellous bone revealed greater energy to ultimate force in RAL versus VEH-D rats, and non-significant trends for greater toughness in RAL rats versus VEH-ND (p = 0.07) and VEH-D (p = 0.06) rats. Ultimate stress was significantly greater in RAL rats compared with VEH-D rats (**Table 3**). VEH-D rats had lower ultimate force and stiffness compared with RAL and VEH-ND, but the corresponding material property of modulus was not different among groups.

**Table 3:** Structure-level and material-level mechanical properties of femoral cortical and cancellous bone and L4 cancellous bone from female ZDSD rats^a^.

	VEH-ND (n = 8)^b^		VEH-D (n = 4)		RAL (n = 12)^c^	
**Femur 3-point bending (cortical bone)**						
*Structure-level*						
Ultimate Force, N	135.1(2.7)	a	135.6 (3.6)	a	142.7 (2.1)	a
Stiffness, N/mm	342.4 (11.1)	a	346.8 (14.7)	a	342.4 (8.5)	a
Energy to Failure, mJ	46.0 (2.9)	a	43.7 (3.8)	a	57.4 (2.2)	b
Post-Yield Energy to Failure, mJ	26.5 (3.0)	a	23.5 (4.0)	a	36.6 (2.3)	b
Post-Yield Displacement, mm	0.210 (0.023)	a	0.187 (0.030)	a	0.275 (0.017)	a
*Material-level*						
Ultimate Stress, MPa	61.4 (1.6)	a	61.3 (2.1)	a	65.0 (1.2)	a
Elastic Modulus, MPa	2683 (92)	a	2724 (122)	a	2691 (70)	a
Toughness, mJ/m^3^	1.21 (0.08)	a	1.13 (0.10)	a	1.51 (0.06)	b
Post-Yield Toughness, mJ/m^3^	0.69 (0.07)	a	0.61 (0.10)	a	0.96 (0.06)	b
**Distal femur RPC (cancellous bone)** b						
*Structure-level*						
Ultimate Force, N	21.5 (2.3)	a	7.3 (3.1)	b	23.7 (1.9)	a
Stiffness, N/mm	239.4 (16.8)	a	142.6 (22.3)	b	249.1 (14.1)	a
Energy to Ultimate Force, mJ	1.41 (0.89)	ab	0.26 (1.18)	a	4.16 (0.75)	b
*Material-level*						
Ultimate Stress, MPa	20.6 (2.5)	ab	13.6 (3.4)	a	27.6 (2.1)	b
Modulus, MPa	400.7 (51.6)	a	474.5 (68.3)	a	534.8 (43.2)	a
Toughness, mJ/m^3^	0.68 (0.55)	a	0.25 (0.73)	a	2.40 (0.46)	a
**L4 axial compression (cancellous bone)**						
*Structure-level*						
Ultimate Force, N	369.9 (18.9)	a	317.3 (25.0)	a	350.4 (15.1)	a
Stiffness, N/mm	1739 (117)	a	1476 (155)	a	1793 (93)	a
Energy to Ultimate Force, mJ	46.6 (3.2)	a	40.9 (4.2)	a	41.1 (2.5)	a
*Material-level*						
Ultimate Stress, MPa	2.27 (0.12)	a	2.11 (0.16)	a	2.22 (0.10)	a
Modulus, MPa	1175 (93)	a	1060 (123)	a	1269 (74)	a
Toughness, mJ/mm^3^	2.51 (0.13)	a	2.28 (0.17)	a	2.31 (0.10)	a

a Different letters in each row indicate differences among groups by Tukey's post-hoc comparisons, p<0.05.

b n = 7 for VEH-ND for distal femur RPC, L4 axial compression, and femur 3-point bending measures, due to specimens breaking during preparation or unavailable sample.

c n = 10 for RAL for the distal femur RPC measures and n = 11 for RAL for L4 axial compression measures, due to specimens breaking during preparation.

## Discussion

This study showed that raloxifene treatment in female ZDSD rats improved blood glucose levels and showed a trend for prevention of type 2 diabetes while imparting a beneficial effect on bone material properties. While the frequency of diabetes between vehicle and raloxifene treated animals was not statistically different by Fisher's exact test, there was a significant difference by survival analysis using Kaplan-Meier curves. This is not conclusive but at least suggestive of a benefit of raloxifene for prevention of diabetes. The finding that raloxifene might prevent the onset of type 2 diabetes in ZDSD rats was an unexpected outcome of this study. A randomized controlled trial [15] found that raloxifene did not improve insulin sensitivity or glycemic control in postmenopausal women who had type 2 diabetes, and a post-hoc analysis [16] of the Multiple Outcomes of Raloxifene Evaluation (MORE) trial found no effect of raloxifene on glycemic control in postmenopausal women with or without diabetes, although a beneficial effect was found on serum lipids. However, these two studies did not evaluate the effect of raloxifene on diabetes onset. Conversely, our results are supported by experimental evidence on the effect of raloxifene on glucose homeostasis and diabetes: it has been shown that estradiol prevents pancreatic β-cell failure in diabetic rats fed a high-fat diet by suppressing fatty acid synthesis and accumulation within the β-cells through estrogen receptor signaling [17], and the same research group found similar results with raloxifene in an *in vitro* study [18]. Therefore, a potential mechanism by which raloxifene could prevent the onset or slow the progression of diabetes is by preventing pancreatic β-cell failure. Additionally, two recent clinical studies [19], [20] found a beneficial effect of raloxifene on serum lipids in women with type 2 diabetes, further supporting a role beyond bone for raloxifene to improve health in people with diabetes.

The fact that none of the raloxifene treated animals became diabetic, while an interesting outcome in itself, was a limitation of this study as it precluded our ability to analyze the effects of raloxifene on bone in rats with established diabetes. However, we were able to show a benefit of raloxifene on bone toughness in a diabetes-prone rat model. While we have previously reported a positive effect of raloxifene on bone toughness in non-diabetic canines [6], [7], this effect has not been previously shown in bones of normal rats [21], [22]. It is possible the predisposition to diabetes in the ZDSD rats creates a therapeutic window for an effect of raloxifene on bone toughness that is not present in normal rats.

Another possible limitation of our study is that we did not include a ZDSD group on a normal diet. However, this would not have been a true control for the diabetes-prone rats because the effects of the different dietary composition on bone's material properties are not known. Moreover, we did not use CD rats which are sometimes used as non-diabetic controls. CD rats are not prone to diabetes even on a high fat diet, but are prone to obesity, and would introduce additional weight-related variables that could affect BMD or other mechanical properties of bone.

The effect of raloxifene on bone toughness was significant only for the femur, representing an effect on cortical bone, but a near-significant trend for greater toughness with raloxifene treatment was also observed for the distal femur by RPC, indicating a possible effect on cancellous bone as well. Our previous canine studies showed a beneficial effect of raloxifene on toughness in both cortical and cancellous bone [6], [7]. Because cortical bone turnover is relatively slow [23], the effect of raloxifene on cortical bone toughness implies a direct effect of raloxifene on the existing bone material, rather than on newly formed bone. Furthermore, intracortical remodeling does not normally occur in rats and does not occur in the ZDSD rats. One mechanism by which raloxifene may improve toughness is by altering the hydration of the bone. We have shown that bone beams carved from human and dog cortical bone, when soaked in a raloxifene solution, have greater toughness and that this is associated with higher water content of the bone [24]. Greater toughness and hydration were also observed in cortical bone beams from dogs treated *in vivo* with raloxifene for 1 year [24].

Additionally, our previous canine study showed no effect of raloxifene on BMD, which corresponds to clinical data from raloxifene trials in which fracture risk is reduced with little change in BMD [8]–[10]. Similarly, in the present study of diabetes-prone ZDSD rats, treatment with raloxifene resulted in greater femoral toughness without an effect on BMD, suggesting that raloxifene affects bone strength by improving bone quality rather than quantity. Because people with type 2 diabetes often have normal or high bone mineral density, the increased fracture risk observed in these patients appears to be due to impaired bone quality rather than reduced bone quantity. However, in this study, diabetic rats actually had lower bone density and mass compared with non-diabetic rats. This difference between human type 2 diabetes and the ZDSD rat model might be explained as follows: in humans with type 2 diabetes, overweight and obesity often persist after the onset of diabetes, and excess body weight may be protective of bone mass through mechanical loading or the positive effects of leptin and estrogen produced by adipose tissue. Conversely, ZDSD rats gain weight with the high fat diet until the onset of diabetes, after which they begin to lose body weight due to the catabolic state produced by the diabetes. Indeed, diabetic rats in the present study had significantly lower body weight at the time of sacrifice compared with non-diabetic animals, and this may be associated with their lower BMD.

The results did not show lower bone resorption as measured by serum CTX in the raloxifene treated rats. However, the numerically lower BFR/BS and MS/BS with raloxifene treatment (−26% and −27% respectively in RAL versus VEH-ND rats) supports that the raloxifene treatment had an effect on reducing bone turnover. The non-significant differences are not surprising given this study was not powered to detect differences in these outcomes, and that raloxifene is a relatively weak antiresorptive agent [25]. However, the magnitude of the difference in BFR/BS with raloxifene treatment is similar to what we previously observed in dogs [7]. Additionally, the rats in this study were not ovariectomized, which also may have reduced our ability to detect a significant antiresorptive effect of raloxifene. The rats that became diabetic (VEH-D) had a lower bone formation rate, which is consistent with reduced bone observed in humans and animals with diabetes [26]. Despite the lack of significant differences in bone turnover measures with raloxifene treatment, our results show that raloxifene improves bone material properties, potentially through direct action of raloxifene on the bone matrix, and may prevent the induction of diabetes in female ZDSD rats. The risk of diabetes increases with age [27], as does the risk for bone fragility fractures [28]. If these results are supported by future experimental and clinical studies, they suggest that raloxifene could be a useful drug to prevent skeletal fragility in diabetes with an added benefit of ameliorating the diabetic condition.

## Supporting Information

Table S1 **Primary data.**
(XLSX)
